# Linking Cultural Tightness, Components of Norm Activation and COVID-19 Preventive Behaviors among University Students: Evidence from Beijing, China

**DOI:** 10.3390/ijerph20064905

**Published:** 2023-03-10

**Authors:** Yang Zou, Xianwei Liu, Miaomiao Yu, Yichu Deng

**Affiliations:** 1 College of Business Administration, Capital University of Economics and Business, Beijing 100070, China; 2 School of Public Administration, Beihang University, Beijing 100191, China; 3 Institute of Higher Education, Beihang University, Beijing 100191, China; 4 Institute of Education Economics and Management, University of Science and Technology Beijing, Beijing 100083, China; 5 College of Holistic Education, Beijing University of Technology, Beijing 100124, China

**Keywords:** cultural tightness, COVID-19 preventive behaviors, norm activation model, university students

## Abstract

The ongoing COVID-19 pandemic has imposed greater challenges and more stringent requirements on higher education institutions (HEIs). However, limited empirical research has been devoted to identifying external and internal factors that may promote individual preventive behaviors during the COVID-19 pandemic within the higher education context. This study proposed and examined an extended norm activation model (NAM) concerning the relationships among cultural tightness, original NAM components, and COVID-19 preventive behaviors. An online survey was conducted with a sample of 3693 university students from 18 universities in Beijing, China. The results showed that cultural tightness was positively associated with respondents’ COVID-19 preventive behaviors. Three original NAM variables, namely, awareness of consequences, the ascription of responsibility, and personal norms, played a chain mediating role in the relationship between cultural tightness and COVID-19 preventive behaviors. Theoretical and practical implications regarding the findings of this study and suggestions for future research are discussed.

## 1. Introduction

The ongoing COVID-19 pandemic caused by a novel coronavirus has been deemed a “once in a century health crisis” and the “worst crisis since World War II” [1]. In the past three years, the rapid spread of the COVID-19 pandemic has brought about continuous and devastating attacks on health, the economy, and livelihood throughout the world [2]. By the end of 2022, COVID-19 had infected over six hundred million people and caused more than 6 million fatalities across 230 countries and areas [3]. Although most governments have started large-scale vaccination programs, considering the high degree of inconsistency of the COVID-19 prevention policies adopted by different countries and the highly variant characteristics of COVID-19, the encouragement of COVID-19 preventive behavior among the public is necessary and vital [4,5].

Higher education institutions (HEIs) faced greater obstacles and stricter regulations related to COVID-19 prevention and management than other social organizations [6]. Due to the high incidence of COVID-19 infection in places with crowded people [7], once one student is infected, widespread pandemic transmission is expected to occur. Under such circumstances, university campuses have been important areas for epidemic prevention. In countries such as China, strengthening the prevention literacy and behaviors of faculty members and students and promoting individuals to consciously abide by epidemic prevention regulations was the focus of a series of schemes and guidelines for preventing and controlling infection in the “new normal” period of COVID-19 before widely approved medicines and vaccines for COVID-19 were developed [8].

Increasing numbers of studies have explored the driving factors of COVID-19 prevention behaviors, while only a few were conducted in higher education systems [4,9,10], and these mainly focused on the role of rational motives from perspectives, such as the Theory of Planned Behavior [4], the Health Belief Model [11], and the Protection Motivation Theory [12]. However, due to the high contagiousness of COVID-19, prevention behaviors not only protect the individual but also have an apparent prosocial and altruistic component [13]. Thus, these behaviors may be dictated by triggering the moral motives of the target population, which the norm activation model (NAM hereafter) emphasizes [5]. In addition, to fight the pandemic, countries or regions that have a culture with higher tightness, such as China, tend to formulate more stringent social norms and intervention strategies to inhibit the spread of COVID-19. Tight culture has been supposed to allow effective control over the number of COVID-19 infections and deaths [14]. Recent research indicates that if China abandons its zero-COVID policies, the country’s healthcare system will almost certainly be overwhelmed, and the highly contagious omicron variant of COVID-19 would likely kill 1.55 million people in China within 6 months [15]. A growing body of cross-cultural research has linked cultural tightness to norm compliance at the individual level [16]. Scholars also noted that tighter societies were better able to regulate people’s behaviors to achieve the collective goal of controlling the virus [14]. However, to our knowledge, limited evidence regarding the effect of tight culture on individual prevention behaviors has been found.

HEIs are regarded as effective settings for shaping specific student attitudes and behaviors through the exportation of culture and social norms [17,18,19]. Based on the theory of planned behavior, our previous study highlighted the critical role of rational motives and institutional factors of HEIs in predicting university students’ COVID-19 preventive behaviors [4]. In this present study, we explicitly detect the cultural and normative antecedents of university students’ COVID-19 preventive behaviors by linking cultural tightness and NAM components within a comprehensive framework. The primary goal of this current study is to explore the association between cultural tightness and the COVID-19 preventive behaviors of university students and examine the chain mediating role of three core NAM components, namely, awareness of consequences, the ascription of responsibility and personal norms, in the relationship between cultural tightness and university students’ prevention behaviors against COVID-19. By doing so, this current study adds to the literature by introducing a prosocial and cultural view of COVID-19 preventive behavior. Specifically, the findings of this study will broaden the NAM’s potential for use in COVID-19 prevention and enhance its explanatory capacity by incorporating extraneous cultural variables. More importantly, with a better understanding of what drives the preventive behaviors of undergraduates, we expect this current study can provide additional insight into anti-epidemic procedures and policies in the HEI setting.

## 2. Theoretical Framework and Hypotheses

### 2.1. Extended NAM and COVID-19 Preventive Behaviors

Schwartz initially formulated the NAM as an altruistic behavior paradigm. The NAM stipulates three exogenous components: awareness of consequences, the ascription of responsibility, and personal norms [20]. The basic assumption of the NAM is that the three components and the targeted behaviors form a causal chain in which each variable directly influences the following one. Since its proposal, the NAM has been supported as an influential framework for understanding behaviors in a wide range of domains, including health-related behaviors [21,22,23,24]. Recent studies focused on explaining individuals’ COVID-19 preventive behaviors clearly reflected that the substantial expansion or modification of the NAM may render it sufficient for explaining COVID-19 preventive behaviors among various populations and across contexts. For example, researchers have assessed variables affecting a series of behaviors toward COVID-19 prevention (e.g., COVID-19 mitigation behaviors, compliance with COVID-19 prevention guidelines, public transport use with COVID-19 precautions, mask-saving behaviors, social distancing compliance, and preventive travel behaviors) by combining the NAM and other prominent theoretical models (e.g., Theory of Planned Behavior, Moral Disengagement Theory, Political Opportunity Structure Theory, Health Belief Model) [25,26,27,28,29].

**Figure 1:**
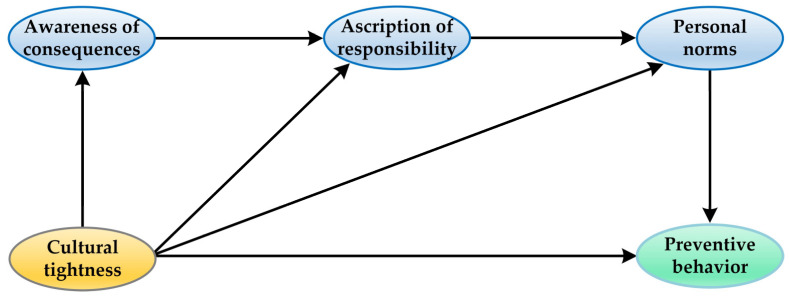
Research framework.

Unlike the Theory of Planned Behavior, which stresses the central role of social norms in predicting behavioral intentions or actual behaviors, NAM emphasizes the importance of personal norms in relation to performing or not performing a certain behavior. Not surprisingly, one recurring criticism of the NAM as a self-interest framework is that it underrepresents the function of external and contextual factors in shaping behaviors [30]. From the viewpoint of social cognitive theory, human behaviors are related to individual factors and social environmental factors [31]. At the social–environmental level, a number of studies have shown the need to include social norms as additional predictors of individual intentions in the NAM [32]. Scholars have highlighted the critical role of social norms, i.e., “what most people typically do or approve of” [33] (p. 397) in motivating a range of health behaviors, including COVID-19 preventive behaviors [34,35]. Social norms vary with regard to the degree of cultural tightness across groups, cities, and societies [36]. Recent studies have examined the influence of cultural tightness, i.e., the strength of social norms on reducing the spread of COVID-19 and case mortality rates [14,37,38]. Their work suggests that tighter groups tend to cooperate faster and more effectively in response to threats than looser groups. Based on the above perspectives and findings, this present study extended the NAM by incorporating cultural tightness as a predictor of both the original NAM variables and COVID-19 preventive behaviors. Figure 1 shows the model in this study.

### 2.2. Cultural Tightness and COVID-19 Preventive Behaviors

Cultural tightness here is defined as university students’ perception of the strength of social norms toward COVID-19 prevention and the degree of tolerance for deviant behaviors from these norms within the society they live in. Since the outbreak and rapid spread of COVID-19, a growing body of literature has mainly focused on linking individual-level cultural tightness with emotional responses and psychological outcomes related to COVID-19. For example, Dong et al. demonstrated that cultural tightness relieves people’s psychological disorders [39]. Liu et al. found that a perceived tighter culture is related to positive psychological outcomes, such as lower pressure, positive emotions, higher trust, and more confidence in combating COVID-19 [40]. Baldner et al. further revealed that the desire for cultural tightness is associated with an increase in individuals’ emotional reactions to noncompliance with preventive measures against COVID-19 [41]. It is worth noting that two recent studies have shed light on the direct effect of cultural tightness on individuals’ COVID-19 preventive behaviors. Specifically, Gilliam et al. found cultural tightness to be positively associated with several self-reported protective health behaviors (e.g., mask wearing, handwashing) during COVID-19 among 544 residents in the U.S. [42]. Based on longitudinal data from an international survey, Schumpe et al. provided empirical evidence for the critical role of the tightness of culture in predicting adherence to recommended health behaviors against COVID-19 [43]. In light of these previous findings, this study proposes the following hypothesis:

**Hypothesis** **1** **(H1).** *Cultural tightness is positively related to university students’ COVID-19 preventive behaviors*.

### 2.3. Mediating Role of NAM Components

Following the definition stated by Schwartz [20], personal norms are university students’ feelings of moral obligation to perform or refuse to engage in COVID-19 preventive behaviors within the context of this study. Based on the perspective of the NAM, personal norms are activated by university students’ awareness of the negative outcomes of not taking COVID-19 preventive behaviors (awareness of consequences) and feelings of responsibility for taking COVID-19 preventive behaviors and preventing the spread of the COVID-19 virus (ascription of responsibility) successively [20,44]. Several recent studies have contributed empirical evidence to support the sequential influence of NAM components on COVID-19 preventive behaviors [5,45,46,47,48]. Indeed, much of the literature has reached a consensus that personal norms are the core of the NAM and the proximal predictor of COVID-19 preventive behaviors and that ascriptions of responsibility and personal norms serially mediate the relationship between awareness of adverse consequences of COVID-19 on individuals’ health and COVID-19 preventive behaviors.

Scholars have noted that cultural tightness helps initiate the causal chain relationship in Schwartz’s original NAM model [49]. Few studies, however, have directly examined the association between individual-level cultural tightness and NAM components in the context of COVID-19 prevention. An exception is a study by Gilliam et al. [42], which deemed that the risk of the COVID-19 pandemic offered a unique chance to evaluate how cultural tightness relates to individual responses to threats and demonstrated that higher levels of perceived tightness were associated with higher reported feelings of responsibility for one’s life. Generally, societies with a tight culture tend to require their citizens to strictly adhere to social norms, thus leading social members to be more willing to comply with rules and to perceive more responsibility for others [14,42,50], which helps protect them from the COVID-19 epidemic and increases the probability that individuals will believe that exhibiting COVID-19 preventive behaviors is necessary and appropriate. In view of the above discourse, it seems very likely that when university students perceive that society maintains strict rules toward COVID-19 prevention in general, it may first trigger the conduction mechanism of NAM components and then enhance their COVID-19 preventive behaviors. Therefore, we propose the following hypotheses:

**Hypothesis** **2** **(H2).** *Personal norms are positively related to university students’ COVID-19 preventive behaviors*.

**Hypothesis** **3** **(H3).** *Cultural tightness is positively related to NAM components*.

**Hypothesis** **4** **(H4).** *NAM components play a chain mediation role between cultural tightness and university students’ COVID-19 preventive behaviors*.

## 3. Methodology

### 3.1. Sample and Data Collection

This study employs data from the COVID-19-specific Module in the second round of the Higher Education and Sustainability Survey (HESS) implemented in 2021. We targeted undergraduates in Beijing because it is the capital city and educational center of China, as well as one of the world’s most populated cities. To ensure the representativeness of the samples, the survey recruited respondents according to the proportions of students in each of the 18 participating universities. Then, the specific colleges or departments were selected using simple random sampling based on the number of respondents assigned to each university. The sample inclusion criteria were undergraduates (excluding international students) who were enrolled full time for at least a full semester (20 weeks) at a single university. In March 2021, soon after the start of spring semester, with the aid of the student affairs staff during the epidemic, Wenjuanxing (a web survey tool in China) survey links were distributed to 4000 undergraduates from all selected colleges or departments in 18 universities. We informed the study’s objectives, the voluntariness and confidentiality of participation, and other issues that participants needed to be aware of in order to complete the questionnaire items on the survey’s instruction page. At the end of April 2021, 3987 questionnaires were collected online. The questionnaires completed in less than 3 min (normal response times were 3–8 min) or with consecutive identical answers in a single scale were regarded as invalid responses. Consequently, a total of 3693 qualified questionnaires were obtained for data analysis after 294 invalid questionnaires were eliminated (see Table 1 for more details about the 3693 respondents’ characteristics). Note, there were no significant differences (*p* > 0.05) in demographic variables were found between the final sample and the original sample according to the results of chi-square tests.

### 3.2. Measures

The questionnaire was divided into two sections. The first section was regarding the background information of the respondents, as shown in Table 1. The second section of the measurement comprised five scales that measured the university students’ perceptions of cultural tightness, awareness of consequences, ascription of responsibility, personal norms, and COVID-19 preventive behaviors. All of the measurement items were adopted from validated scales of existing studies or government publications and were accompanied by a five-point Likert scale. With the assistance of two bilingual experts working at the corresponding author’s university, the original English version scales of the awareness of consequences, ascription of responsibility, and personal norms were translated into Chinese using a translation and back-translation technique.

A six-item Chinese version of the cultural tightness/looseness scale [37,50] was adapted to assess the respondents’ perceived cultural tightness in the context of COVID-19 prevention. University students rated each item on a 5-point Likert scale ranging from 1 (strongly disagree) to 5 (strongly agree), with higher scores indicating a higher level of perception of social norm strength and strictness toward COVID-19 prevention.

For the NAM components, the awareness of consequences scale and ascription of responsibility scale were revised by Yazdanmehr and Wang [51]. The 3-item awareness of consequences scale asks respondents to rate their agreement with each statement about the adverse consequences of violating preventive measures on a 5-point Likert scale ranging from 1 (strongly disagree) to 5 (strongly agree). The ascription of responsibility scale comprises 2 items asking respondents to indicate their agreement with each statement about the responsibility for preventing the COVID-19 epidemic on a 5-point Likert scale ranging from 1 (strongly disagree) to 5 (strongly agree). A 3-item personal norms scale was adapted from Si et al. [25] to assess the respondents’ moral obligation to exhibit COVID-19 prevention behaviors on a 5-point Likert scale ranging from 1 (strongly disagree) to 5 (strongly agree).

A seven-item COVID-19 preventive behavior scale was developed from the officially recommended preventive measures of the Chinese Center for Disease Control and Prevention by Liu et al. [52]. We asked the university students to rate the frequency with which they had performed each of 6 preventive actions during the COVID-19 period on a 5-point Likert scale ranging from 1 (never) to 5 (always).

### 3.3. Data Analysis

We followed the two-step strategy suggested by Anderson and Gerbing to test both the reliability and validity of the measurement model and to examine the hypothesized relationships in this study via structural equation modeling (SEM) with a maximum-likelihood estimation approach [51]. Both absolute fit indices and incremental fit indices were adopted to evaluate the goodness-of-fit of the model. The former included ratio of chi-square to the degree of freedom (*χ*^2^/*df* ≤ 5), goodness-of-fit index (GFI ≥ 0.90), standardized root mean square residual (SRMR < 0.08), and root mean square error of approximation (RMSEA < 0.08). The latter included incremental fit index (IFI ≥ 0.90), comparative fix index (CFI ≥ 0.90), and Tucker–Lewis index (TLI ≥ 0.90). However, given that *χ*^2^/*df* was sensitive to sample volume [52], and when all the 3693 responses are involved in the analysis, the values of GFI, RMSEA, SRMR, IFI, CFI, and TLI may present a more rational and appropriate criterion to judge model fit [53]. We further ran a bootstrapping analysis with 2000 iterations to generate bias-corrected estimates of the indirect influences of cultural tightness on COVID-19 preventive behavior (via the chain mediation of awareness of consequences, ascription of responsibility, and personal norms) and their corresponding 95% confidence intervals (CIs). If a 95% CI for an indirect effect does not cover 0, it can be concluded that the mediation effect tested is statistically significant at the 0.05 level. All statistical analyses were processed based on the Amos 26 statistical package.

## 4. Results

### 4.1. Descriptive Statistics

The results of descriptive statistical analysis indicated that (see Table 2 for more details) the mean scores of the constructs and specific items varied from 3.821 to 4.699, the standard deviation varied from 0.529 to 0.894, the absolute coefficients of skewness were between 0.049 to 1.857 (lower than the threshold level of 3), and the absolute coefficients of kurtosis lied between 0.224 to 7.209 (lower than the threshold level of 10), supporting that the 5 constructs and their items fulfilled the approximately normal distributions, and the subsequent analysis could be executed [53].

### 4.2. Measurement Model

**Figure 2:**
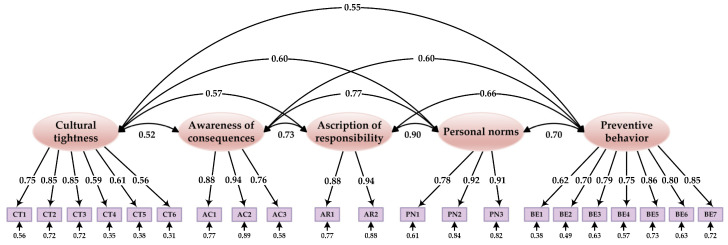
Results of the CFA.

We first conducted a confirmatory factor analysis (CFA) to confirm the fitness of the measurement model to the research data and to provide validity and reliability information about the constructs before structural model assessment. The measurement model comprised 5 latent constructs and 21 observed items. In the CFA, we allowed the latent constructs to correlate with each other, whereas the observed indicators were stipulated to load only on their respective constructs. The results of CFA revealed that all fit indexes met the threshold requirements for adequate fit except the value of *χ*^2^/*df* (*χ*^2^ = 3112.495; *df* = 179; *χ*^2^/*df* = 17.388; GFI = 0.914; CFI = 0.948; IFI = 0.948; TLI = 0.939; SRMR = 0.041; RMSEA = 0.067 [90% CI: 0.065, 0.069]). However, given the large sample size of this study, we still deemed the model fit acceptable. As shown in Figure 2, the standardized factor loadings of all items were significant and ranged from 0.556 to 0.942 (greater than the threshold level of 0.50) [54].

We performed Harman’s single-factor test to detect the potential common method variance in the survey data [55]. We compared the fit of the 1-factor model (the common method) to the hypothesized 5-factor model and found that the 1-factor model (all 21 items were restricted to load on a single latent construct) failed to provide a good fit to the data (*χ*^2^ = 17669.232; *df* = 189; *χ*^2^/*df* = 93.488; GFI = 0.599; CFI = 0.689; IFI = 0.689; TLI = 0.654; SRMR = 0.101; RMSEA = 0.158 [90% CI: 0.156, 0.160]. The chi-square statistic (Δ*χ*^2^ = 14,556.737, Δ*df* = 10, *p* < 0.001) also indicated that the measurement model better fit the data than the 1-factor model, thus ruling out the possibility of common method bias in this present study.

After the CFA, we further assessed the reliability and validity of the constructs. As seen in Table 3, Cronbach’s α coefficients for the 5 constructs varied from 0.845 to 0.905 (higher than the recommended value of 0.700) [54]. The values of composite reliability (CR) were between 0.856 and 0.910 (higher than the threshold standard of 0.7), indicating an acceptable level of internal consistency [56]. Additionally, the average variance extracted (AVE) values ranged from 0.506 to 0.828 (greater than the benchmark value of 0.40), demonstrating satisfactory convergent validity for all constructs [57].

As shown in Table 4, all of the correlation coefficients among variables were significant and showed that the expected directions ranged from 0.449 to 0.825 (*p* < 0.001). The significance of the variables’ correlations satisfied the basic requirements for mediation analysis recommended by Baron and Kenny [58]. Moreover, as the square roots of the AVEs for all of the constructs were greater than their correlations with each other, the discriminant validity of the measurement was also established [57].

### 4.3. Structural Model

**Figure 3:**
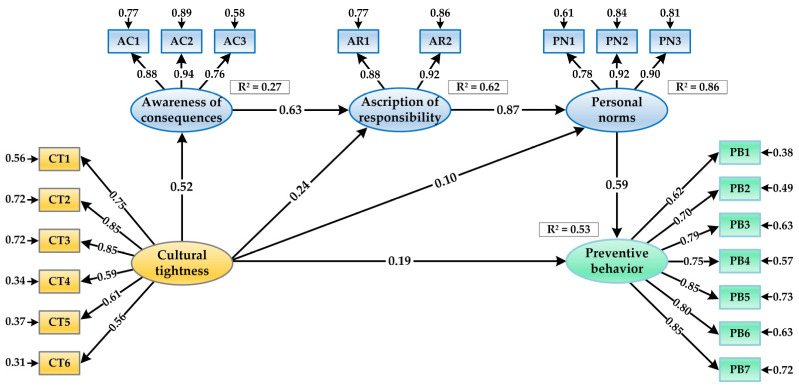
Results of the SEM analysis.

We implemented SEM analysis to assess the relationships hypothesized in the structural model. The results indicated that the posited structural model fit the data adequately (*χ*^2^ = 3372.860; *df* = 182; *χ*^2^/*df* = 18.532; GFI = 0.908; CFI = 0.943; IFI = 0.943; TLI = 0.934; SRMR = 0.046; RMSEA = 0.069 [90% CI: 0.067, 0.071]). Then, we calculated the direct path coefficients and their statistical significance level. As illustrated in Figure 3, all direct effects in the structural model were statistically significant. First, the direct link between cultural tightness and students’ COVID-19 preventive behaviors was significant (*β* = 0.194, *t* = 10.574, *p* < 0.001). Second, cultural tightness was positively associated with all 3 original NAM components, namely, awareness of consequences (*β* = 0.523, *t* = 24.590, *p* < 0.001), the ascription of responsibility (*β* = 0.243, *t* = 14.857, *p* < 0.001), and personal norms (*β* = 0.099, *t* = 7.558, *p* < 0.001). Finally, we found that personal norms had a positive and significant relationship with the students’ COVID-19 preventive behaviors (*β* = 0.594, *t* = 31.072, *p* < 0.001). Thus, H1, H2, and H3 were supported. In addition, the results revealed that the structural model could explain 53% of the variance in students’ COVID-19 preventive behaviors.

A bootstrapping analysis was further conducted to test the mediation effects in the hypothesized model. As seen in Table 5, both the direct and indirect relations between cultural tightness and COVID-19 preventive behavior were significant, suggesting that awareness of consequences, the ascription of responsibility, and personal norms played a chain mediating role in the association between cultural tightness and students’ COVID-19 preventive behaviors. Therefore, H4 was verified.

## 5. Discussion and Implications

With an expanded NAM framework, this study aimed to identify the critical linkage between cultural tightness and COVID-19 preventive behaviors and the serial mediation effect of three original NAM components in this relationship among university students in Beijing, China. The results of our study provided empirical evidence for all of the relationships hypothesized in the research framework, and the key findings are outlined and discussed as follows.

Our study confirmed a significant positive relationship between individual-level cultural tightness and the COVID-19 preventive behaviors of university students. This finding implies that when university students of this sample perceive their society to be tighter, they are more likely to adopt health behaviors against COVID-19; in contrast, those who perceive their society to be less tight will be less prone to comply with the recommended COVID-19 protective behaviors. Apart from mapping global differences, scholars have directed much attention to the variation in cultural tightness within a single country [59,60]. A large-scale study of cultural tightness–looseness indicated that Beijing, among 31 provinces and cities in China [61], could be classified as a tight city, and university students in Beijing were more willing or more pressured to abide by concrete or potential social norms toward COVID-19 prevention. Under the strict zero-COVID-19 policy, students’ deviations from COVID-19 prevention measures were more likely to incur severe punishment due to the greater accountability pressure faced by HEIs in containing the rapid spread of COVID-19. Therefore, students would have expected greater acceptance and compliance with regulations or recommendations for health behaviors during the COVID-19 pandemic [14]. Our finding is consistent with recent studies conducted in Western countries that vary in terms of the level of cultural tightness. For example, researchers found that respondents from the US, Canada, UK, and Australia who reported being tighter were more willing to comply with recommendations for protective behaviors against COVID-19 [42,62].

As expected, we demonstrated that personal norms are the nearest and strongest predictor of university students’ COVID-19 preventive behaviors and cooperate with the other two NAM variables to form a mediation chain in the relationship between cultural tightness and COVID-19 preventive behaviors. This result provides critical empirical evidence for the assumption that cultural tightness contributes to starting the causal–consecutive link between original NAM variables and individual behaviors [49]. A possible reason may be that cultural tightness is too general to guide the actual preventive behaviors of university students [63]. Thus, it should be first converted to personal norms through an internalization procedure; namely, individuals should be aware of the consequences of not performing COVID-19 preventive behaviors and feel responsible for the adverse consequences of not performing these behaviors. Accordingly, it can be concluded that a city or institution with a tight culture generally emphasizes strict norms or regulations against COVID-19 infection, which may lead to a high level of COVID-19 preventive behaviors by triggering the internal awareness and responsibility necessary for university students’ moral obligations to flourish.

Our findings make the following theoretical contributions. First, our research fills a void in the literature by drawing upon the perspectives of cultural psychology and the prosocial behavior paradigm, thus providing a comprehensive framework for understanding COVID-19 preventive behavior within the context of higher education. Second, although existing studies have highlighted the critical role of cultural tightness in combating the COVID-19 pandemic at the global and national levels [14,37], there is a relatively small body of literature concerned with the individual level. Our study explicitly detected the direct effect of cultural tightness on university students’ COVID-19 preventive behaviors and offered a response to the call for further evaluation of the potency of cultural tightness in influencing the degree of adherence to COVID-19 prevention requirements [64]. Third, this study attempted to overcome the limitation of the original NAM by considering the influence of contextual or external variables on behaviors. To the best of our present knowledge, this is the first study to establish empirical support for the chain mediating role of NAM core elements in connecting COVID-19 preventive behaviors and external factors, for example, the cultural tightness considered in this study.

We provided two major suggestions for authorities and HEIs to encourage the implementation of preventive actions among young people in the “new normal” period of containing the COVID-19 virus. First, given the fundamental role of cultural tightness in motivating COVID-19 preventive behaviors, tightening social norms may be one of the most effective ways to coordinate attitudes and behaviors among students when they confront urgent and collective threats from the COVID-19 pandemic [65,66]. Despite this perspective and our findings, we and other scholars would like to recommend that governments and HEIs adopt balanced strategies that emphasize both togetherness and social order in regions with tight cultures (e.g., the “United and determined, we shall prevail” and “When an epidemic breaks out, a command is issued”, an anti-COVID slogan adopted frequently on many university campuses in China) [67]. Second, due to the pro-social nature of COVID-19 preventive behaviors and the mediation mechanism of NAM variables in bridging cultural tightness and preventive behaviors, educational authorities and HEIs should strengthen students’ perceptions of the severity of the COVID-19 pandemic and the consequences of not complying with COVID-19 prevention requirements on the wellbeing of others through educational intervention, thus activating their personal moral obligations to better adhere to specific restrictions and increase voluntary efforts to combat the COVID-19 pandemic in the long run.

## 6. Conclusions

In summary, in this study, we showed quantitative evidence that cultural tightness not only influences COVID-19 preventive behaviors directly but also exerts an indirect effect through the chain mediation of awareness of consequences, the ascription of responsibility, and personal norms in a sample of university students in Beijing, China. Hence, the NAM-based expansion model proposed in this study could be considered a promising framework for investigating the external and internal factors leading to university students’ preventive behaviors on campus.

Notwithstanding the aforementioned implications, there were some limitations that should be considered in future work. Importantly, as the findings of this study are based on cross-sectional design and self-reported data collected from university students in Beijing, the capital city of China, it may be somewhat risky to generalize them to university students in other areas or cities in China. Therefore, we recommend that future studies consider longitudinal study design and within-nation differences in cultural tightness [61,68], thus offering more accurate cross-cultural evidence or adjusting the research models used to explain the role of cultural and normative factors in driving individual COVID-19 preventive behaviors. In fact, in a recent study conducted in the US, Gilliam and colleagues revealed that individuals’ ratings of their states’ tightness rather than these states’ archival scores on tightness were associated with positive attitudes and behaviors toward COVID-19 prevention [42]. Moreover, this study was conducted approximately one year after the outbreak of the COVID-19 pandemic. Thus, these data may not reflect how the evolution of the COVID-19 pandemic affected China’s anti-COVID-19 policies or the attitudes, norms, and behaviors of students. For example, on 7 December 2022, China announced a nationwide loosening of COVID-19 restrictions, in which PCR testing would be reduced, and lockdowns would also be limited. Among the changes, the health pass application will no longer be required for entry to most public spaces, and patients with mild symptoms may quarantine at home rather than in facilities [69]. However, the unexpected loosening was followed by a sharp infection surge, which has aroused widespread confusion, fear, and anxiety among the public; meanwhile, most people have reduced their spontaneous social activities and performed more frequent preventive behaviors against infection [70]. Hence, new investigations should be designed to further consider the interactive and complex relationships among cultures, policies, and social psychology on individuals’ COVID-19 preventive behaviors.

## Figures and Tables

**Table 1:** Characteristics of respondents (*N* = 3693).

Variable	Group	*n*	*%*
Gender	Female	1857	50.3
	Male	1836	49.7
Major	Science and Engineering	2782	75.3
	Humanities and Social Sciences	911	24.7
Grade	Freshman	1231	33.3
	Sophomore	897	24.3
	Junior	839	22.7
	Senior	726	19.7
Ethnicity	Han	3305	89.5
	Other	388	10.5

**Table 2:** Scale items and descriptive statistics.

Variables/Measurement Items	Mean	SD	Skewness	Kurtosis
**Awareness of consequences**	4.495	0.581	−1.009	1.173
AC1: I believe violation of the preventive measures will cause serious trouble to the public.	4.540	0.630	−1.614	4.532
AC2: I believe violation of the preventive measures will cause serious damage to the campus.	4.535	0.619	−1.323	2.613
AC3: I worry about serious problems caused by violations of the preventive measures.	4.412	0.677	−1.094	1.619
**Ascription of responsibility**	4.433	0.613	−0.873	1.133
AR1: I feel responsible to do something against the spread of the COVID-19 epidemic.	4.433	0.646	−1.044	1.759
AR2: I feel responsible to help those around me comply with the preventive measures.	4.434	0.636	−0.987	1.557
**Personal norms**	4.442	0.570	−0.656	0.282
PN1: I would feel guilty for not complying with the preventive measures.	4.338	0.725	−1.246	2.575
PN2: Adopting the preventive measures is consistent with my moral principles.	4.461	0.595	−0.747	0.527
PN3: I feel morally obligated to comply with the preventive measures.	4.527	0.560	−0.766	0.331
**Preventive behaviors**	4.500	0.529	−0.935	0.761
BE1: Minimize social activities; avoid infected areas; avoid crowded public places.	4.361	0.746	−1.225	1.924
BE2: Wear a single-use medical face mask when visiting public places or taking public transport.	4.699	0.548	−1.927	4.487
BE3: Keep your hands clean and wash your hands frequently; minimize contact with objects in public places.	4.470	0.666	−1.102	1.103
BE4: Refrain from touching your mouth, nose, and eyes with unwashed hands; cover your mouth and nose with your elbow when sneezing or coughing.	4.404	0.746	−1.265	1.726
BE5: Monitor your health conditions and comply with campus epidemic prevention regulations.	4.596	0.575	−1.275	1.801
BE6: Ensure your home is adequately ventilated.	4.463	0.687	−1.220	1.553
BE7: Maintain distance from others in public places to reduce unnecessary infections.	4.509	0.649	−1.247	1.665
**Cultural tightness**	4.113	0.575	−0.162	0.102
CT1: There are many social norms that people are supposed to abide by in this country.	4.384	0.615	−0.654	0.572
CT2: In this country, there are very clear expectations for how people should act in most situations.	4.287	0.669	−0.745	0.990
CT3: People agree on what behaviors are appropriate versus inappropriate in most situations in this country.	4.236	0.685	−0.847	1.740
CT4: People in this country have a great deal of freedom in deciding how they want to behave in most situations. (Reverse coded)	3.957	0.894	−0.879	0.749
CT5: In this country, if someone acts in an inappropriate way, others will strongly disapprove.	3.989	0.797	−0.584	0.381
CT6: People in this country almost always comply with social norms.	3.821	0.890	−0.620	0.290

**Table 3:** The standard factor loading of items and the reliability of the scales.

Variables	Items	Loading	Cronbach’s α	CR	AVE
Cultural tightness (CT)	CT1	0.749	0.845	0.856	0.506
	CT2	0.848			
	CT3	0.851			
	CT4	0.588			
	CT5	0.613			
	CT6	0.556			
Awareness of consequences (AC)	AC1	0.879	0.888	0.898	0.747
	AC2	0.942			
	AC3	0.763			
Ascription of responsibility (AR)	AR1	0.880	0.905	0.906	0.828
	AR2	0.939			
Personal norms (PN)	PN1	0.779	0.887	0.903	0.756
	PN2	0.917			
	PN3	0.906			
Preventive behaviors (BE)	BE1	0.619	0.904	0.910	0.593
	BE2	0.700			
	BE3	0.794			
	BE4	0.752			
	BE5	0.855			
	BE6	0.795			
	BE7	0.846			

**Table 4:** Square root of AVEs and correlations between constructs.

Variables	1	2	3	4	5
1. Cultural tightness	*0.711*				
2. Awareness of consequences	0.449 ***	*0.864*			
3. Ascription of responsibility	0.489 ***	0.685 ***	*0.910*		
4. Personal norms	0.514 ***	0.715 ***	0.825 ***	*0.869*	
5. Preventive behaviors	0.467 ***	0.561 ***	0.596 ***	0.633 ***	*0.770*

Diagonal elements (in italics) are the square root of the AVEs; *** *p* < 0.001.

**Table 5:** Results of bootstrapping analysis.

Paths	Bootstrapping		95% Bias-Corrected CI	
	Effect	Boot S. E.	Boot LLCI	Boot ULCI
CT → PB	0.194 ***	0.020	0.155	0.233
PN → PB	0.594 ***	0.021	0.553	0.634
CT→ AC	0.523 ***	0.019	0.484	0.559
CT → AR	0.243 ***	0.024	0.198	0.292
CT → PN	0.099 ***	0.018	0.066	0.139
CT → AC → AR → PN → PB	0.185 ***	0.015	0.158	0.217

*** *p* < 0.001.

## Data Availability

The data used or analyzed during this current study are available from the corresponding author on request.
